# Supplementary material for: Application of novel PACS-based informatics platform to identify imaging based predictors of CDKN2A allelic status in glioblastomas
Source: Sci Rep. 2023 Dec 22;13:22942. doi: 10.1038/s41598-023-48918-4 (PMC10746716; doi:10.1038/s41598-023-48918-4)
Supplement: Supplementary file 1 — Supplementary Information. [file 41598_2023_48918_MOESM1_ESM.docx]

**Supplementary data:**

**Table 1)**

| VASARI Item | Answers | Score |
| --- | --- | --- |
| Hemorrhage | Yes  No | 45  24 |
| Calvarial remodeling | Yes  No | 1  68 |
| Cysts | Yes  No | 1  68 |
| Tumor Location | Frontal  Temporal  Insular  Parietal  Occipital  Brainstem  Cerebellum | 21  25  -  17  4  1  1 |
| Side of Tumor Epicenter | Right  Center/Bilateral  Left | 36  2  31 |
| Eloquent Brain | None  Speech motor  Speech receptive  Motor  Vision | 27  14  19  4  4 |
| Multifocal or Multicentric | n/a  Multifocal  Multicentric  Gliomatosis | 65  2  1  1 |
| T1/FLAIR RATIO Tumor | Expansive  Mixed  Infiltrative | 69  0  0 |
| Pial Invasion | Yes  No | 41  28 |
| Ependymal Invasion | Yes  No | 55  14 |
| Cortical Involvement | Yes  No | 60  9 |
| Deep WM Invasion | Yes  No | 47  22 |
| Satellites | Yes  No | 10  59 |
| Lesion Size | <0.5 cm  0.5 cm  1.0 cm  1.5 cm  2.0 cm  2.5 cm  3.0 cm  3.5 cm  4.0 cm  4.5 cm  5.0 cm  5.5 cm  6.0 cm  6.5 cm  7.0 cm  7.5 cm  8.0 cm  > 8.0 cm | -  -  -  1  -  2  1  3  3  4  4  4  3  2  3  2  3  34 |
| Proportion of Edema | n/a  None (0%)  <5%  6-33%  34-67%  68-95%  >95%  All (100%)  Indeterminate | -  2  1  7  30  29  -  -  - |
| Edema Crosses Midline | n/a  Yes  No | 1  3  65 |
| Enhancement Quality | None  Mild/Minimal  Marked/Avid | 1  3  65 |
| Proportion Enhancing | n/a  None (0%)  <5%  6-33%  34-67%  68-95%  >95%  All (100%)  Indeterminate | -  1  -  1  25  32  6  3  1 |
| Thickness of Enhancing Margin | n/a  None  Thin  Thick/solid | -  1  1  67 |
| Definition of the Enhancing Margin | n/a  Well-defined  Poorly-defined | 1  64  4 |
| Enhancing Tumor Crosses Midline | n/a  Yes  No | 4  2  63 |
| Proportion non Contrast Enhancing | n/a  None (0%)  <5%  6-33%  34-67%  68-95%  >95%  All (100%)  Indeterminate | -  69  -  -  -  -  -  -  - |
| Proportion Necrosis | n/a  None (0%)  <5%  6-33%  34-67%  68-95%  >95%  All (100%)  Indeterminate | -  3  8  32  23  1  -  -  2 |
| Definition of the non-enhancing margin (e.g. Grade III) | n/a  Smooth  Irregular | 2  11  56 |
| Diffusion | No Image  Facilitated  Restricted  Neither/equivocal | 8  5  55  1 |
| nCET Tumor Crosses Midline | n/a (nonCET)  Yes  No | 2  6  61 |

**Table 1)** All scorable VASARI items in our analysis with their respective scores


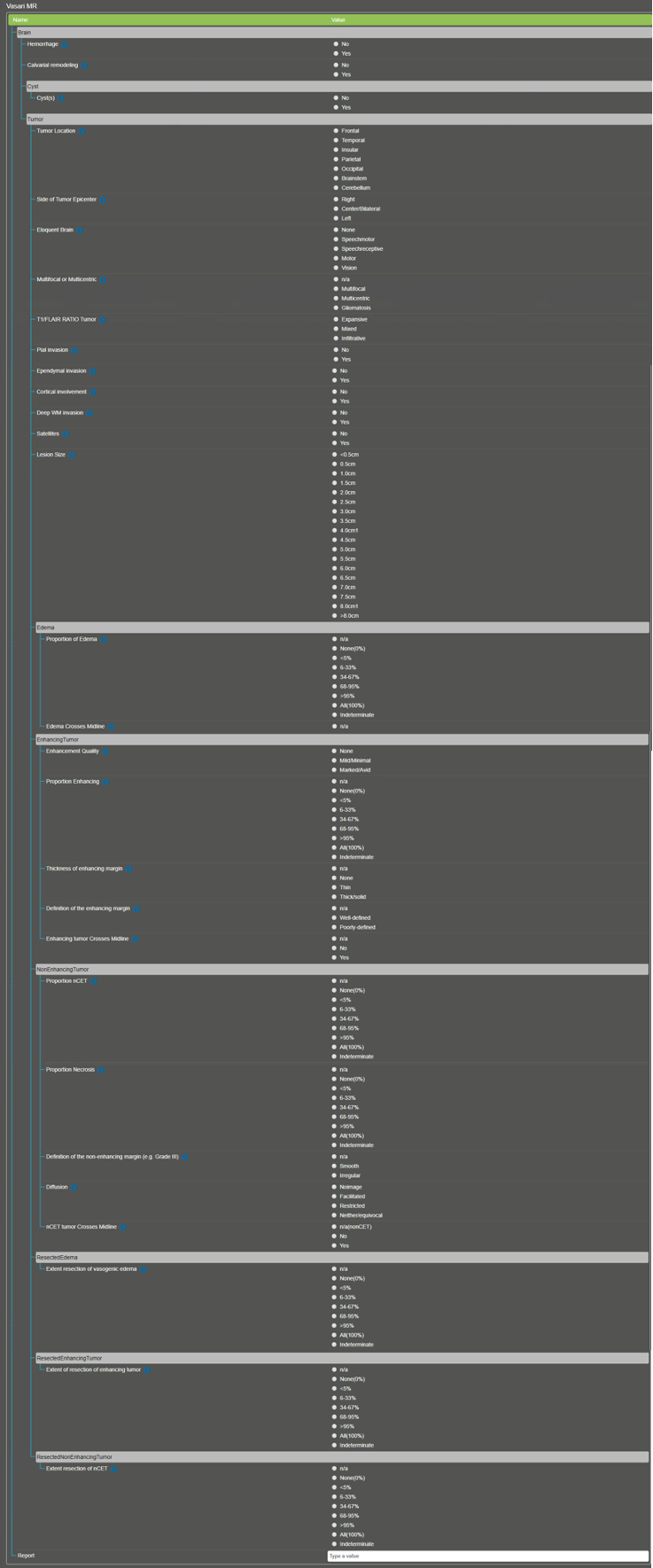


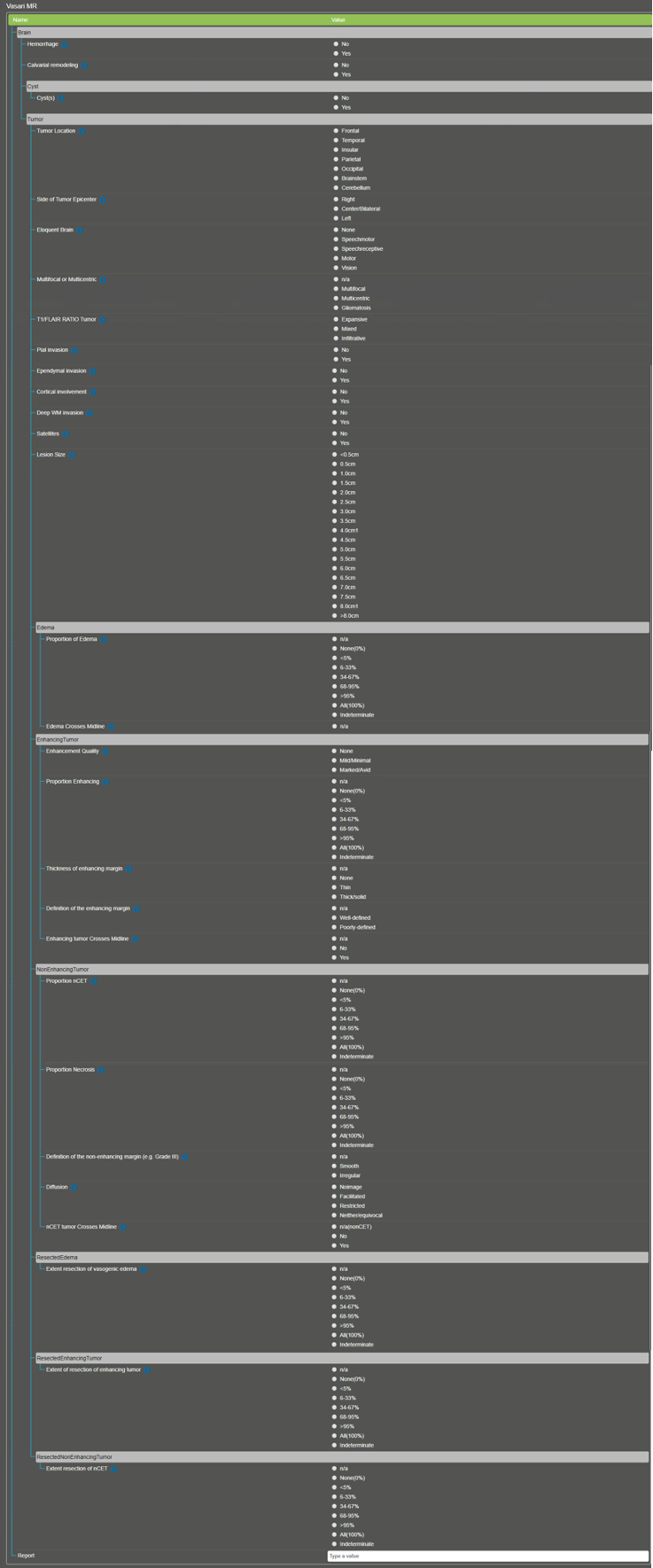


**Figure 1)** Layout of the PACS embedded FHIR form containing the VASARI questionnaire

| VASARI items | Definition according to VASARI form | No. of patients | CDKN2A  HETLOSS  (N=25) | CDKN2A  HOMDEL  (N=17) | CDKN2A  No alteration  (N=27) | P values |
| --- | --- | --- | --- | --- | --- | --- |
| Proportion of Edema | 0-33% | 10 | 4 (16%) | 1 (5.9%) | 5 (18.5%) | 0,59 |
|  | 34-67% | 30 | 13 (52%) | 7 (41.2%) | 10 (37%) |  |
|  | 68-95% | 28 | 8 (32%) | 9 (52.9%) | 11 (40.7%) |  |
|  | Unknown^1^ | 1 | 0 (0%) | 0 (0%) | 1 (3.7%) |  |
| Proportion Enhancing | 0-67% | 27 | 11 (44%) | 5 (29.4%) | 11 (40.7%) | 0,632 |
|  | 68-100% | 40 | 14 (56%) | 12 (70.6%) | 14 (51.9%) |  |
|  | Unknown | 2 | 0 (0%) | 0 (0%) | 2 (7.4%) |  |
| Proportion Necrosis | 0-33% | 42 | 15 (60%) | 11 (64.7%) | 16 (59.3%) | 0,946 |
|  | 34-100% | 24 | 10 (40%) | 5 (29.4%) | 9 (33.3%) |  |
|  | Unknown | 3 | 0 (0%) | 1 (5.9%) | 2 (7.4%) |  |
| Hemorrhage | Yes | 45 | 15 (60%) | 12 (70.6%) | 18 (66.7%) | 0,683 |
|  | No | 23 | 10 (40%) | 4 (23.5%) | 9 (33.3%) |  |
|  | Unknown | 1 | 0 (0%) | 1 (5.9%) | 0 (0%) |  |
| Tumor location | Brainstem | 1 | 1 (4%) | 0 (0%) | 0 (0%) | 0,429 |
|  | Cerebellum | 1 | 1 (4%) | 0 (0%) | 0 (0%) |  |
|  | Frontal | 21 | 6 (24%) | 3 (17.6%) | 12 (44.4%) |  |
|  | Occipital | 4 | 1 (4%) | 2 (11.8%) | 1 (3.7%) |  |
|  | Parietal | 17 | 7 (28%) | 6 (35.3%) | 4 (14.8%) |  |
|  | Temporal | 25 | 9 (36%) | 6 (35.3%) | 10 (37%) |  |
| Side of Tumor Epicenter | Center/Bilateral | 2 | 2 (8%) | 0 (0%) | 0 (0%) | 0,266 |
|  | Left | 31 | 8 (32%) | 8 (47.1%) | 15 (55.6%) |  |
|  | Right | 36 | 15 (60%) | 9 (52.9%) | 12 (44.4%) |  |
| Eloquent Brain | Motor | 4 | 3 (12%) | 0 (0%) | 1 (3.8%) | 0,463 |
|  | Vision | 4 | 1 (4%) | 2 (13.3%) | 1 (3.8%) |  |
|  | Speech motor | 13 | 4 (16%) | 1 (6.7%) | 8 (30.8%) |  |
|  | Speech receptive | 18 | 6 (24%) | 6 (40%) | 6 (23.1%) |  |
|  | None | 27 | 11 (44%) | 6 (40%) | 10 (38.5%) |  |
| Pial invasion | Yes | 41 | 14 (56%) | 14 (82.4%) | 13 (48.1%) | 0,072 |
|  | No | 28 | 11 (44%) | 3 (17.6%) | 14 (51.9%) |  |
| Ependymal invasion | Yes | 55 | 22 (88%) | 10 (58.8%) | 23 (85.2%) | 0,062 |
|  | No | 14 | 3 (12%) | 7 (41.2%) | 4 (14.8%) |  |
| Cortical involvement | Yes | 59 | 21 (84%) | 15 (88.2%) | 24 (88.9%) | 0,858 |
|  | No | 10 | 4 (16%) | 2 (11.8%) | 3 (11.11%) |  |
| Deep WM invasion | Yes | 50 | 17 (68%) | 8 (47.1%) | 22 (81.48%) | 0,058 |
|  | No | 19 | 8 (32%) | 9 (52.9%) | 5 (18.52%) |  |
| Satellites | Yes | 10 | 5 (20%) | 1 (5.9%) | 4 (14.8%) | 0,472 |
|  | No | 58 | 19 (76%) | 16 (94.1%) | 23 (85.2%) |  |
|  | Unknown | 1 | 1 (4%) | 0 (0%) | 0 (0%) |  |
| Lesion Size | <=8.0cm | 35 | 16 (64%) | 11 (64.7%) | 8 (29.6%) | 0,02 |
|  | >8.0cm | 34 | 9 (36%) | 6 (35.3%) | 19 (70.4%) |  |
| Definition of the non-enhancing margin | Irregular | 56 | 20 (80%) | 13 (76.5%) | 23 (85.2%) | 0,704 |
|  | Smooth | 11 | 3 (12%) | 4 (23.5%) | 4 (14.8%) |  |
|  | Unknown | 2 | 2 (8%) | 0 (0%) | 0 (0%) |  |
| Diffusion | Facilitated | 5 | 3 (12%) | 0 (0%) | 2 (7.4%) | 0,591 |
|  | Neither/equivocal^2^ | 1 | 0 (0%) | 0 (0%) | 1 (3.7%) |  |
|  | No Image | 8 | 3 (12%) | 1 (5.9%) | 4 (14.8%) |  |
|  | Restricted | 55 | 19 (76%) | 16 (94.1%) | 20 (74.1%) |  |
| nCET tumor Crosses Midline | Yes | 6 | 3 (12%) | 0 (0%) | 3 (11.1%) | 0,372 |
|  | No | 61 | 21 (84%) | 17 (100%) | 23 (85.2%) |  |
|  | Unknown | 2 | 1 (4%) | 0 (0%) | 1 (3.7%) |  |
| OSF (Outside Facility) | OSF | 10 | 2 (8%) | 4 (23.5%) | 4 (14.8%) | 0,428 |
|  | Yale | 58 | 22 (88%) | 13 (76.5%) | 23 (85.2%) |  |
|  | Unknown | 1 | 1 (4%) | 0 (0%) | 0 (0%) |  |
| Ethnicity | White | 61 | 22 (88%) | 15 (88.2%) | 24 (88.9%) | 1 |
|  | Other | 8 | 3 (12%) | 2 (11.8%) | 3 (11.1%) |  |
| Sex | Female | 25 | 6 (24%) | 8 (47.1%) | 11 (40.7%) | 0,246 |
|  | Male | 44 | 19 (76%) | 9 (52.9%) | 16 (59.3%) |  |
| Age at Surgery | Mean |  | 61 | 62 | 64 | 0,78 |
|  | Standard deviation |  | 18 | 11 | 15 |  |

**Table 2)** Descriptive statistics of qualitative radiomic features summarized by the 3 subgroups of CDKN2A. Based on the distributions of these features, we classified CDKN2A subgroups, and conducted statistical testing to investigate the differences in the features between the reclassified subgroups. For the correlations between subgroups and features, Fisher’s Exact Test was used for categorical variables, while Student’s t test or Mann-Whitney U test was used for continuous variables based on the distribution.

(^1^Note: Unknowns were excluded from calculating p values ^2^Neither/equivocal was excluded in the analysis of correlation)
